# Raman Imaging with a Fiber-Coupled Multichannel Spectrograph

**DOI:** 10.3390/s141121968

**Published:** 2014-11-20

**Authors:** Elmar Schmälzlin, Benito Moralejo, Monika Rutowska, Ana Monreal-Ibero, Christer Sandin, Nicolae Tarcea, Jürgen Popp, Martin M. Roth

**Affiliations:** 1 Leibniz-Institut für Astrophysik Potsdam (AIP), An der Sternwarte 16, Potsdam 14482, Germany; E-Mails: bmoralejo@aip.de (B.M.); mrutowska@aip.de (M.R.); ana.monreal-ibero@obspm.fr (A.M.-I.); csandin@aip.de (C.S.); mmroth@aip.de (M.M.R.); 2 GEPI Observatoire de Paris, CNRS, Université Paris Diderot, Place Jules Janssen, Meudon 92190, France; 3 Institute of Physical Chemistry, Helmholtzweg 4, Jena 07743, Germany; E-Mails: nicolae.tarcea@uni-jena.de (N.T.); juergen.popp@ipht-jena.de (J.P.); 4 Leibniz Institute of Photonic Technology, Albert-Einstein-Straße 9, Jena 07745, Germany; 5 Institut für Physik und Astronomie, Universität Potsdam, Karl-Liebknecht-Str. 24-25, Potsdam 14476, Germany

**Keywords:** multichannel Raman spectroscopy, astronomy spectrograph, optical fiber bundle, Raman imaging

## Abstract

Until now, spatially resolved Raman Spectroscopy has required to scan a sample under investigation in a time-consuming step-by-step procedure. Here, we present a technique that allows the capture of an entire Raman image with only one single exposure. The Raman scattering arising from the sample was collected with a fiber-coupled high-performance astronomy spectrograph. The probe head consisting of an array of 20 × 20 multimode fibers was linked to the camera port of a microscope. To demonstrate the high potential of this new concept, Raman images of reference samples were recorded. Entire chemical maps were received without the need for a scanning procedure.

## 1. Introduction

Laser Raman spectroscopy [1] is a powerful technique to determine chemical compositions. In combination with a microscope, high contrast chemical maps of the sample under investigation can be constructed. The Raman spectrum contains information about the intrinsic vibration levels of chemical compounds. Such spectral fingerprints can be used to identify certain molecules or chemical groups without labeling. There are many applications for Raman images, for example the inspection of pharmaceutical ingredients [2], detection of cancerous tissue [3], examination of crystalline structures [4], monitor drug distribution within cells [5], and the molecular imaging of cells [6]. A review of various modern Raman techniques, their use in microscopy and further examples of use can be found in [7]. Usually, a Raman image is recorded by scanning the sample spatially point by point. Other scanning techniques have been implemented using point/line scanning or continuous scanning coupled with different ways of synchronizing detector read-out and sample scanning. Broad laser beam illumination coupled with tunable band-pass filters have also been used for quick Raman imaging. The scanning procedure, however, has a severe disadvantage: Due to the small Raman scattering cross section, and consequently generally very low intensities of the Raman signal, for most samples even modern spectrometers usually need at least a few seconds to capture the signal at any single point. As a result, the recording of an entire Raman image may take up to several hours even at modest resolution. Such long data acquisition periods are not suitable for most applications, e.g., for medical *in situ* examinations of a patient, or for monitoring chemical reactions in real time. Furthermore, a step-by-step scanning procedure requires high-precision mechanics to shift either the sample or the focus of the excitation laser beam, which again is not practical for e.g., a clinical environment. Finally, long time measurements require high stabilities of laser source and optical components.

Therefore, first experiments were devised to employ spatial multiplexing techniques in order to replace the cumbersome scanning procedure by an exposure of many spatially resolved elements simultaneously in “one shot” [8,9]. The multiplexing can be achieved e.g., by using a fiber bundle to sample the optical image of an extended source on one end, and feeding a rearranged linear array of the fibers at the output of the bundle to a spectrograph. The early experiments, however, were severely hampered by the limited field-of-view of standard laboratory spectrographs, hence a narrow free spectral range (∼600 cm^−1^) and a disappointingly small number of spatial elements (<t;70) [8], or by low sensitivity of the instrument [9].

**Figure 1.**
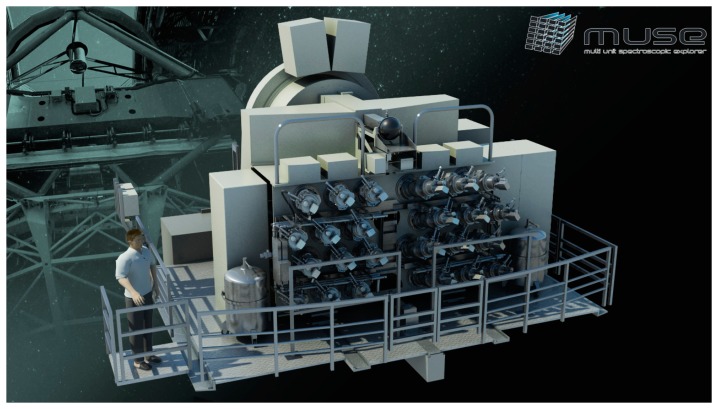
Layout of MUSE at the ESO VLT at Paranal Observatory, Chile (credit: CRAL, Lyon, France).

In contrast, a similar problem in modern astrophysics, namely spatially resolved spectroscopy of faint extended galaxies, has been solved by introducing the method of Integral-Field Spectroscopy (IFS) already more than two decades ago. First generation highly efficient integral field spectrographs have provided many spectra of order several hundreds to thousands over a full 2-dimensional field-of-view simultaneously in a single exposure [10]. Today, IFS is an established common-user technique at all major observatories. Second generation IFS instruments, e.g., VIRUS for the Hobby Eberly Telescope in Texas [11], or MUSE (Multi Unit Spectroscopic Explorer) for the Very Large Telescope at the ESO Paranal Observatory in Chile [12], provide more than one order of magnitude more spatial elements. MUSE e.g., delivers a total of 90,000 individual spectra, resulting in spectral images of 300 × 300 spatial elements (“spaxels”). This instrument covers a free spectral range over one octave from 465 nm to 930 nm with a spectral resolution of ∼0.25 nm and unprecedented sensitivity. In order to master the extremely high multiplex gain and, consequently, the complexity of the instrument, a modular design was adopted. It incorporates, amongst various other subsystems, a total of 24 highly efficient identical spectrographs. MUSE was designed to discover the faintest galaxies of the universe that would be invisible by other methods. Very high efficiency is accomplished by an optimized high-throughput and low aberration refractive collimator-camera system, a volume phase holographic grating, and a graded-index AR-coating, liquid nitrogen-cooled 4096 × 4112 pixel CCD detector (CCD231, e2v, Saint-Egrève Cedex, France). A conceptual sketch of the instrument, that was commissioned at Unit Telescope 4 of the VLT in March 2014 and has undergone science verification in June 2014 [13], is shown in Figure 1.

As an attempt of technology transfer, we have adapted the concept of IFS to laboratory Raman spectroscopy by making an ad hoc combination of a fiber array with a copy of the high-end MUSE unit spectrograph, to record entire images without scanning [14]. In this paper, we present the first results that demonstrate the feasibility of this concept.

## 2. Experimental Section

### 2.1. MUSE Spectrograph and Fiber Bundle Probe Head

**Figure 2.**
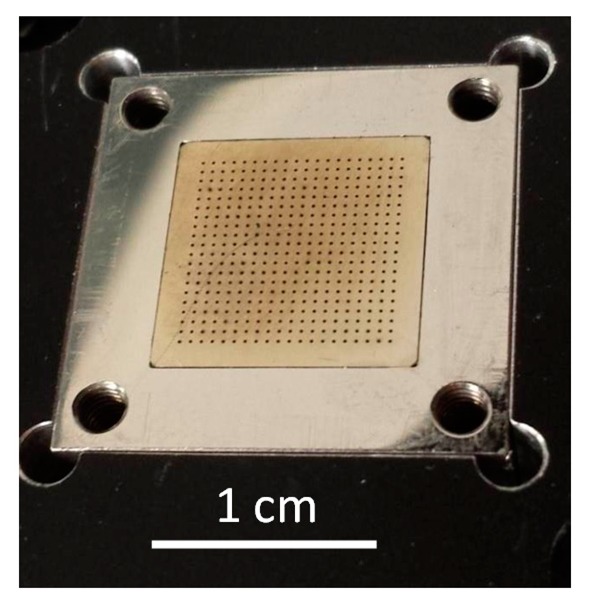
The probe head with 20 × 20 fibers optics matrix. Every black dot represents the front surface of the multimode fiber.

The spectrograph, which was used to detect the Raman signal, is the 25th copy of the MUSE unit spectrographs. In the MUSE configuration, the imaging capability is achieved with a sophisticated image slicer in front of the collimator. However, in our laboratory experiment the MUSE spectrograph was linked to a bundle of 400 110/132 μm step index multimode Fibers (Leoni FiberTech, Neuhaus-Schierschnitz, Germany) instead of the slicer. To form a probe head for recording images, the front surfaces of these fibers are arranged in a 20 × 20 matrix. Figure 2 shows the probe head as viewed face on. The pitch between the fiber centers was 0.5 mm, resulting in a square active surface with 9.5 mm edge length. At the other end of the bundle, the fibers were arranged in one row to form a pseudo-slit for the spectrograph. The dimension of the row is designed to match the first collecting lens of the spectrograph collimator. It must be stressed that the fiber-collimator interfacing was less than optimal as the spectrograph is designed for slicers and anamorphic foreoptics module [15]. That said, the ad hoc combination worked surprisingly well.

The signals of all 400 fibers generate light traces (spectra) at the CCD that are modulated by the intensity distribution across the fiber array. A custom-developed open source data-reduction software, called *p3d*, was used to process the raw CCD image [16]. The software and the calibration procedures were described previously [17–19]. In brief: To subtract zero level signals of the CCD, a master bias with 0 s exposure time was recorded. To identify the light trace positions of each fiber at the CCD and to determine fiber-to-fiber sensitivity variations, the probe head was exposed to the flat signal arising from an integrating sphere, which in turn was linked to a continuum halogen lamp. For wavelength calibration, the integrating sphere was linked to a neon lamp. The software also cleans the data from cosmic ray hits. Finally, the *p3d* software converts the CCD image into a three-dimensional cube, where the first two dimensions contain the spatial information of the image, and the third dimension contains the spectral information at each spatial position.

### 2.2. Free-Space Optical Setup

**Figure 3.**
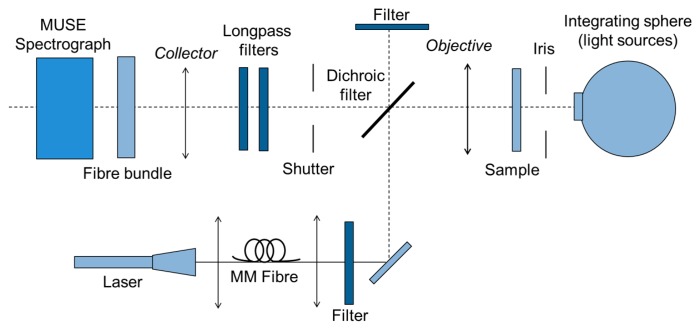
Free-space optical setup to verify the suitability of the MUSE spectrograph for measuring Raman signals.

Figure 3 shows a free-space optical setup, which was built to validate the usefulness of the MUSE spectrograph for Raman spectroscopy. As an excitation source, a 500 mW, 785 nm laser diode coupled with a 600 μm fiber was used. A 785 nm clean-up filter removed side bands of the laser emission. A 785 nm dichroic razor edge beam splitter directed the laser beam to the sample and let the stokes-shifted Raman signal pass. To remove the Rayleigh line, two 785 nm razor edge long pass filters were used. A 20× magnification, numerical aperture (NA) 0.4, IR-optimized objective (Mitutoyo lens) collects the Raman signal and guides the signal to the probe head.

### 2.3. Microscope Setup

**Figure 4.**
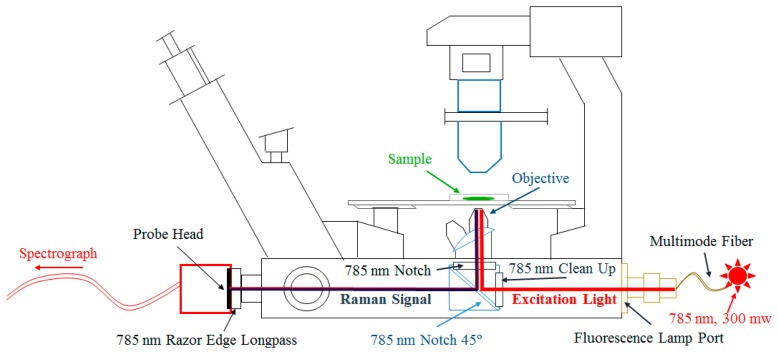
Microscope-based setup for imaging Raman spectroscopy. Here, a schematic drawing of the body of the microscope.

To improve the imaging quality, a second setup with use of a microscope was realized. The probe head with the fiber array was placed at the camera output of an inverted fluorescence microscope (Axiovert.A1 FL, Zeiss, Jena, Germany). Figure 4 shows a scheme of microscope setup. A fiber-coupled 300 mW 785 nm diode laser from a commercially available Raman spectrometer (i-Raman, B<mp;W Tek, Newark, DE, USA) was used as excitation light source. The laser light was coupled to the microscope via the fluorescence lamp port using a 105/125 μm multimode fiber and a collimator. The fluorescence filter cube of the microscope contained a 785 nm clean-up filter, a 785 nm notch beam splitter, and a 785 nm notch filter as emission filter. Additionally, a 785 nm long-pass filter was placed in the front of probe head. The microscope was equipped with 2.5× and 20× magnification objectives, which allow the capture of object fields with 10.0 and 1.2 mm diameters, respectively.

## 3. Results and Discussion

### 3.1. Measurements and Results Achieved with the Free-Space Setup

**Figure 5.**
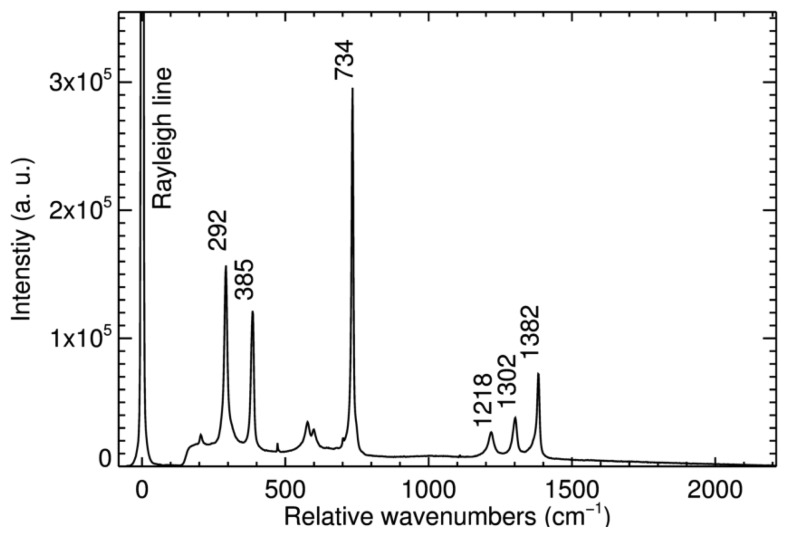
Raman spectrum of a Teflon sample.

Initially, the free-space setup was used with a piece of Teflon, a paracetamol tablet, and a silicon wafer. Figure 5 presents the collected Raman spectrum of Teflon. The curve represents the total signal of all 400 fibers. The exposure time was 60 s. The peaks at 292 cm**^−^**^1^ and 385 cm**^−^**^1^ (torsional and deformation vibrations of CF_2_), 734 cm**^−^**^1^ (symmetric CF_2_ stretching), 1218 cm**^−^**^1^ (antisymmetric CF_2_ stretching), 1302 cm**^−^**^1^ and 1382 cm**^−^**^1^ (C-C stretching), match the values specified in the literature [20]. This consistence proves that the MUSE spectrograph is generally suitable for Raman spectroscopy. In addition, the Raman spectra received with the free-space setup for paracetamol and silicon match the literature values (data not shown) [21,22].

**Figure 6.**
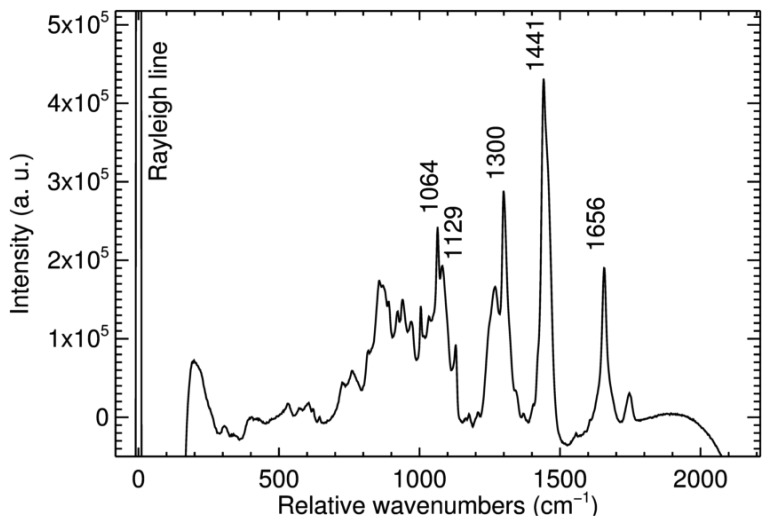
Raman spectrum of a pork sample.

The detection of cancerous tissue is a potential future application [3]. As a tentative experiment, a piece of raw pork meat was examined, since the Raman signals of pork meat are expected to be similar to those of future tissue samples. Figure 6 shows the received Raman spectrum with well identifiable Raman signals. Again the curve represents the total signal of all 400 fibers. The exposure time was 240 s. Fluorescence background was reduced by use of a fourth-degree polynomial fit. The most intense signal peaks are observed at 1656 cm**^−^**^1^, 1441 cm**^−^**^1^, 1300 cm**^−^**^1^, and 1064 cm**^−^**^1^. 1656 cm**^−^**^1^ and 1300 cm**^−^**^1^ can be related to Amide I-helix and Amide III-helix, respectively [23]. 1441 cm**^−^**^1^ is linked to CH_3_, CH_2_, and CH groups, 1064 cm**^−^**^1^ to C-N and C-C bounds.

**Figure 7.**
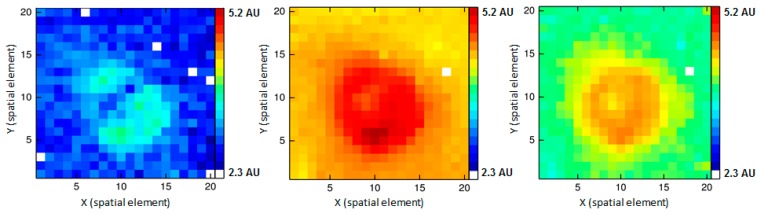
Intensity distribution of Raman signals of pork meat at the front surface of the probe head, (**left**) for 1129 cm**^−^**^1^; (**middle**) for 1449 cm**^−^**^1^; (**right**) for 1659 cm**^−^**^1^.

Figure 7 shows the intensity distributions at certain Raman shifts at the position of the probe head. Although the free space setup was not optimized for imaging, patterns of a first Raman map could be observed. To receive the respective Raman signal intensities, the peaks at 1129 cm**^−^**^1^, 1449 cm**^−^**^1^, and 1659 cm**^−^**^1^ were fit to Gaussian functions and converted into pseudo colors. In Figure 7 the red color represents the highest and blue the lowest signal intensity. White spots indicate damaged fibers, which did not provide an analyzable signal.

The simple disk-shaped intensity distribution is most likely due to the inhomogeneous intensity distribution of the excitation laser and to the shape of the sample, while Figure 7 suggests a more complex distribution, related to structures of the sample. However, with the given limitations we decided to use a microscope for any further measurements as the optical pathway is already optimized with regard to imaging.

### 3.2. Measurements and Results Achieved with the Microscope Setup

We investigated: (I) A mixture of 50 μm polystyrene beads (PS; Acros Organics, Geel, Belgium) and 100 μm polymethyl methacrylate microbeads (PMMA; Acros Organics, Geel, Belgium) microbeads; (II) A sample made up from a half aspirin tablet (Paracetamol BC, Berlin Chemie, Berlin, Germany) and a half paracetamol tablet (ASS-ratiopharm 500, Ratiopharm, Ulm, Germany). The halves were polished and pushed together in a way that the boundary surface passed the center of the object field; (III) A combination drug painkilling tablet containing 50 mg caffeine, 200 mg paracetamol and 250 mg aspirin (Neuradinal N, Stada, Bad Vilbel, Germany). (I) was placed on a small metal plate. The beads stuck to the metal surface even it was turned upside down; (II) and (III) were positioned over the hole of a perforated dish. This allowed a direct illumination without any cover slip in the light path.

The exposure time was 120 s. For subtraction of background arising from optic components of the microscope, reference measurements without sample were performed. The intensity of the excitation laser light was inhomogeneously distributed at the sample surfaces. At the center, the signal intensity was about one order of magnitude higher than at the corners. To moderate the impact of the inhomogeneous laser intensity distribution within the object field, the intensities of the Raman signals of (I), (II), and (III) were normalized by the signal intensity arising from the CaF_2_ plate. To identify the locations of the Raman-active substances, for samples (I), (II), and (III), one characteristic signal peak for each substance, *i.e.*, a peak which shows no or only slight overlap to the peaks of the other incorporated substances, was chosen. Table 1 shows the Raman shifts of the selected peaks specified in wavenumbers.

**Figure 8.**
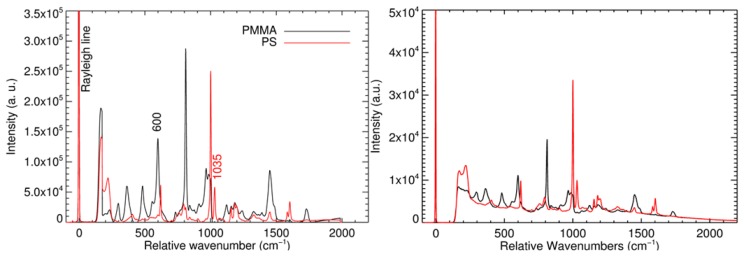
Raman spectra of PS (red line) and PMMA (black line). The heights of the peaks at 600 cm**^−^**^1^ and 1035 cm**^−^**^1^, respectively, were used to identify PS and PMMA. (**Left**) Background-corrected total spectra of PS and PMMA, received by adding up the signals arising from the positions of PS and PMMA beads, respectively; (**Right**) Unprocessed spectra received from two single fibers at the image positions of a PMMA and a PS beads, respectively (X, Y = 8, 9 and X, Y = 6, 12; compare Figure 9).

The heights of each characteristic peak, which resulted after the normalization procedures, were taken as measures of the substance concentrations. Figure 8 shows the Raman spectra received from sample (I). Left part: Added up and background-corrected Raman spectra of PS (red line; total intensity from 15 fibers positioned at the most intense regions of the image of the PS beads) and PMMA (black line; total intensity of 32 fibers positioned at the most intense region of the image of the PMMA bead). Right part: Two spectra from two single fibers as they were received from the spectrograph without further processing. The spectra are in accordance with literature values [24,25]. To remove background fluorescence, an automated algorithm for subtraction was used for all spectra. The chosen method is based on the modified polynomial curve fitting of Lieber and Mahadevan-Jansen [26] fitting the background with a fourth order polynomial retaining the minimum intensity value at each wavenumber at each iteration using a total of 15 iterations per spectrum.

**Figure 9.**
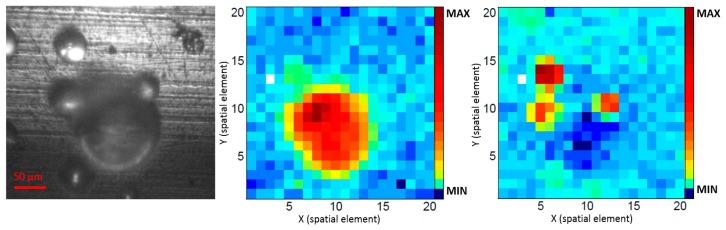
Sample (I); plastic microbeads on a metal plate. (**Left**) image taken with an eye piece camera and a 20× objective; (**Middle**) Raman image of PMMA (600 cm**^−^**^1^); **(Right**) Raman image of PS (1035 cm**^−^**^1^).

**Figure 10.**
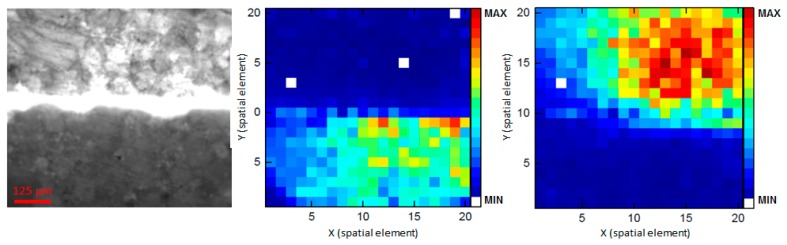
Two half pieces of paracetamol and aspirin pain reliever tablets, respectively, which have been pushed together. (**Left**) image taken with an eye piece camera and a 2.5× objective. Middle: Raman image of aspirin (800 cm**^−^**^1^); (**Right**) Raman image of paracetamol (752 cm**^−^**^1^).

**Figure 11.**
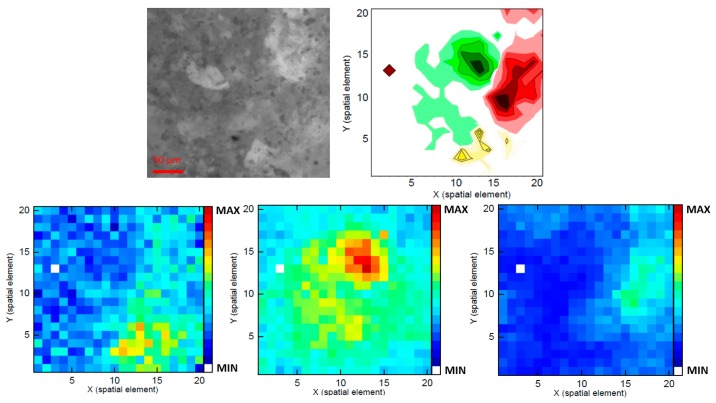
Sample (III): pain reliever tablet with caffeine, aspirin, and paracetamol. (**Upper left**) camera image taken with a 20× objective; (**Upper right**) Overlay of the detected locations of caffeine (yellow), aspirin (green), and paracetamol (red) as contour color illustration. The brown diamond indicates the position of a defective fiber within the probe head. **(Lower left**) Raman image of caffeine (1703 cm**^−^**^1^); (**Lower middle**) Raman image of aspirin (1047 cm**^−^**^1^); (**Lower right**) Raman image of paracetamol (860 cm**^−^**^1^).

To receive chemical Raman maps, the spatial intensity distributions at 600 cm^−1^ (characteristic peak for PMMA) and 1035 cm^−1^ (characteristic peak for PS) were plotted as pseudo colors. Figures 9, 10 and 11 show the Raman maps in comparison to the corresponding camera pictures.

The camera picture of sample (I) (Figure 9, left) shows one big sphere (*ca.* 120 μm diameter) and three smaller spheres (*ca.* 50 μm diameter) in immediate vicinity. A bit further afar, there are four more beads. Three of them are only half within the image field. The spot at the upper right is an anomaly of the surface of the metal plate. The corresponding Raman maps (Figure 9, middle and right) identify the big sphere as PMMA and the small ones as PS. The chemical map corresponds to the camera image. The PS Raman image may suggest a further sphere at the upper left, which is out of the camera field. This is due to slightly smaller image area of the camera in comparison of the area which is covered by the probe head. In spite of the normalization procedure, the PS spheres in the image center clearly show a stronger signal compared to the spheres at the border. The Raman map of the PMMA bead is somewhat cross sensitive to signal of the PS beads. This can be explained by a certain overlap of the PMMA and PS Raman spectra at 600 cm**^−^**^1^ (Figure 8).

Figure 10 left shows the camera image of the sample made of two half pieces of pain relievers (II). The upper tablet half contains paracetamol as active pharmaceutical ingredient, the lower half aspirin. The received Raman maps (Figure 10, middle and right) again clearly correspond to the camera image, even if there seems to be some signal overlapping in the right part of the maps. This is likely due to overlaps of the Raman spectra and some defocusing because of the rough and uneven surfaces of the tablets.

Figure 11 shows the results received from sample (III). The Raman map allows discrimination between regions dominated by paracetamol and dominated by aspirin. The accordance with the camera image is only moderate. On the one hand, surely fillers codetermine the structures of the camera image, on the other hand, substances, which are located below the surface and therefore cannot be seen within the camera image, will contribute to the signal composition. To illuminate the entire image field, the excitation laser is only moderately focused, which in turn causes a loss of resolution within the *z*-axis.

## 4. Conclusions and Outlook

We have presented, to the best of our knowledge, the first validation of astronomical IFS as a tool for creating Raman maps simultaneously from a single exposure. The test was performed with an existing high performance MUSE spectrograph, however, that is not optimized for fiber-coupled Raman spectroscopy [15], meaning that the demonstrated performance must be considered a lower limit, and that there is room for optimization. While the initial free-space optics experiment was merely demonstrating the ability of taking good signal-to-noise Raman spectra, the measurements performed with the microscope clearly prove the Raman imaging capability of the setup. Although the uniformity of the sample illumination was not ideal, all Raman images recorded with the microscope setup are consistent with the camera image. This proves the high potential of this method. To receive information about the gain in time, preliminary comparison measurements with a commercial available Raman microscope (alpha 300 R, Witec, Ulm, Germany) were performed recently. Applying the same excitation intensities per area and the same spatial resolution, Raman images were received more than ten times faster with the MUSE setup. It is worth mentioning that much intensity is wasted when the whole sample area is illuminated. The probe head has a total surface of 90 mm^2^, however, the total area of the fiber core front surfaces measures only 4 mm^2^. As a consequence, only about 4% of the image signal is detected. Ongoing work to improve the sample illumination is in progress. The use of a microlens array as realized in [9] is very promising. We believe that the recording speed can be further improved within the existing setup. However, we are currently developing a derivative of the MUSE spectrograph that is optimized for a fiber-coupled collimator input, also featuring a nominal wavelength range that is extended towards the blue for future Anti-Stokes experiments. These activities are aiming at the future development of label-free spatially resolved real-time monitoring of (bio-)chemical reactions.

## Figures and Tables

**Table 1.** Raman shifts of characteristic signal peaks, which were used to identify the respective substances.

**Specimen**	**PS**	**PMMA**	**Paracetamol**	**Aspirin**	**Caffeine**
Sample (I)	1035 cm^−1^	600 cm^−1^			
Sample (II)	-	-	800 cm^−1^	752 cm^−1^	-
Sample (III)	-	-	860 cm^−1^	1047 cm^−1^	1703 cm^−1^
